# WHERE IS THE DIGITAL DIVIDE? INTRA-GENERATIONAL DIVIDE ON APP USAGE IN POST-PANDEMIC HONG KONG

**DOI:** 10.1093/geroni/igad104.3696

**Published:** 2023-12-21

**Authors:** Bobo Hi-po Lau, Eric Ngai Yin Shum, Fan Yin-Fong Lui, Gigi Lam, Alex Pak-Ki Kwok, Chung-Kin Tsang

**Affiliations:** Hong Kong Shue Yan University, Hong Kong, Hong Kong; Hong Kong Shue Yan University, Hong Kong, Hong Kong; Hong Kong Shue Yan University, Hong Kong, Hong Kong; Hong Kong Shue Yan University, Hong Kong, Hong Kong; The Chinese University of Hong Kong, Hong Kong, Hong Kong; Hong Kong Shue Yan University, Hong Kong, Hong Kong

## Abstract

The COVID pandemic has evoked extensive digitalization of our daily lives. This paper reports two studies conducted in the June/July 2023 that evaluated smartphone usage among Hong Kong middle-aged and older adults after 13 months of smartphone-based compulsory track-and-trace COVID policy. A focus group study with 145 adults aged 55 years or above (Max = 92) found that smartphones are used primarily for instant but asynchronous communication, thus, WhatsApp and WeChat are the most popular. Divisive usage is the most salient with apps that involve monetary transactions (e.g., mobile banking, online shopping) with participants having more trust towards the security provided by the device, the network, and the financial institutions as well as higher socio-economic status (SES) being more receptive. Higher SES was also related to a broader repertoire of app use. These findings corroborate with those from a concurrent survey with 750 smartphone users aged 45 or older (Max = 78). Using latent class analysis, we found two classes of users based on their usage on 18 categories of apps. Class 1 (64.3%) were more likely to use all categories of apps than Class 2 (35.7%), with the largest Chi-square values reported on apps for small-sum transactions, online shopping, private hire cab/courier, and mobile banking – all related to monetary transactions. Class 1 were younger, with higher SES, owning more smart devices, more proficient with mobile devices, and more optimistic with technologies than Class 2. Our findings elucidate directions for narrowing the digital divide within the older adult population.

